# Supplementary material for: Accelerating prediction of chemical shift of protein structures on GPUs: Using OpenACC
Source: PLoS Comput Biol. 2020 May 13;16(5):e1007877. doi: 10.1371/journal.pcbi.1007877 (PMC7250467; doi:10.1371/journal.pcbi.1007877)
Supplement: Attachment — Submitted filename: Author_Responses_PCOMPBIOL-D-20-00060.pdf [file pcbi.1007877.s002.pdf]

April 08, 2020

Dear Dr. Schneidman-Duhovny

We thank you and the reviewers very much for taking the time to send us your detailed feedback and comments. Our responses in this cover letter are inlined in blue.
We have also color coded in red, the new text in the revised PDF.

Please let us know if there are any further questions.

Thank you once again.

Stay safe and take care
Sunita Chandrasekaran
Asst. Prof. UDelaware, US
Corresponding Author
PCOMPBIOL-D-20-00060

Reviewer's Responses to Questions

Comments to the Authors:
Please note here if the review is uploaded as an attachment.

Reviewer #1: This work presents a systematic report that accelerates the PPM_One program by implementing portable optimization using OpenACC. It includes a detailed description of the process that helped improve the efficiency of the PPM_One orders of magnitude. It will greatly benefit the NMR communities and structural biology work involving NMR methods. The manuscript is nearly ready for publication, in my opinion. Here are some minor comments:

1. Page 2-3, Section "Overview of the Scientific Problems: Chemical shift Prediction: The citations in the first three paragraphs do not show up corrected. Please correct them.

Thank you. We have fixed them.

2. Page 6, the end of paragraph 4: The speed-up will be discussed in more detail in Section 3.2. However, sections are not numbered, no idea which this refers to.

This was an oversight. We have fixed this to reflect the right section (Results).

3. Page 7, first sentence of paragraph 2: The function getani() represents the compute region for calculating the chemical shift due to magnetic anisotropy. This sentence may need revision.

This is a great point. We have rewritten for the narrative to flow better

4. Table 1. The times for 6.8m atoms are longer than 5m atoms, which is easy to understand. However, why is it longer than 11.3m atoms? Any particular reason? Would love to see the explanation. Or maybe this reveals there is something else needs optimization?

Thank you for this question. This boils down to two main reasons: 1) the preprocessing step of the data is by far the most time consuming part after acceleration. We made a handful of significant improvements to it, but without completely rewriting it we are limited on how much further we can take it. Currently, the data preprocessing varies quite a bit between different structures. 2) the 'gethbond' function does present a significant difference depending on the structures we worked on. One structure could spend only a few milliseconds in 'gethbond', while others could spend a few seconds. The 'gethbond' function works with the hydrogen atoms in the structure, and for protein structures solved with x-ray crystallography or cryogenic electron microscopy, hydrogen atoms may not be present at all (most often, they are not present for these types of structures). If there are no Hydrogen atoms, then there are no Hbonds to detect and that 'gethbond' function is inactive. These are some of the reasons for the differences in run-time.

Reviewer #2: The manuscript at high level is very well organized and written. This work will definitely help other MD application developers think of using / adapting to programming models such as OpenACC to accelerate their applications, without having to delve into the nitty-gritty's of CUDA.

Minor revisions needed in the abstract and throughout the paper, comments indicated in the pdf (please open using Adobe Reader or similar).

Thank you, yes we expanded NMR, re-worded the abstract, fixed the references. The figures, as per the submission instructions, were not to be included into the main PDF, so they were additional files attached to the manuscript.

Comments:
1. Be explicit in the abstract that there was major refactoring on the cpu side of the code happened as well before accelerating the code to the GPUs. Just 14hrs to 46secs seems like the code was really really bad, and the comparison is not appropriate. Recommend tweaking the statement a bit to reflect the changes (note: this was clear in the results section but not in the abstract).

Thanks and we have fixed the wording to fix this ambiguity in the abstract.

2. Some citations are missing, Fig 1 was missing (I think at least, not in the pdf that was uploaded), also recommend running the manuscript through Grammarly (or similar) for grammatical error fixes.

Yes, the citations have been fixed. Apologies for the oversight. We also ran the manuscript thru grammar check tool via latex (and did spot a few errors and fixed them). The figures are attached as additional files as per the instructions on the template.

3. Are their plans of running such simulations / modeling on larger systems with a higher (and complex) node count and what do the authors think of as a challenge when running similar workloads with OpenACC + MPI (or OpenMPI) ? Maybe something along these lines in a future work, or ongoing work directions would be an appropriate addition to this manuscript.

From the simulations that we did, we did not think that the addition of MPI would be a huge benefit to performance as the overhead presented via MPI would likely take as much time as what would be saved by splitting up the computation. MPI+OpenACC would definitely be an approach on structures that are orders of magnitude bigger than what we have done such as in the case of a future scenario where the experimental techniques like solid-state NMR (which are good for large protein complexes) continue to progress. A multi-node implementation with MPI would enable accelerated PPM in different types of workflows, for instance: validating a large-scale MD simulation with predicted chemical shifts. To make the most out of a massively parallel machine, enabling chemical shift prediction across multiple nodes with MPI would be of interest.

Another technical limitation that the code has now is that it does not immediately present itself to proper data distribution. For example, get_contact() is a function of complexity NxN (where N is our data size), this means that for every data-point, it must be compared to every other data-point. That means that a function like get_contact would be difficult to distribute over a cluster without having the total problem size loaded on all GPUs of all nodes. It would need a handful of rewrites to work. Having said that if the scenario presents itself, then this would be the direction to go.

4. Recommend outlining the challenges of accelerating and porting such an application using OpenACC in a subsection, this will help other MD app developers to take that leap.

Thank you for this thought. We made an attempt to add this blurb into the manuscript but we landed up going way over the word limit. In order to avoid cutting off more essential paragraphs, we moved this blurb into the project's GitHub wiki page and here is the link to it. https://github.com/UD-CRPL/ppm_one/wiki

Reviewer #3: The manuscript describes a significant improvement on the run time of a chemical shift prediction program.

Chemical shift prediction is an important area of ongoing research to leverage the large amount of data available from NMR experiments of protein. The manuscript does a very good job of addressing the case of solid-state NMR, which can tackle large protein assemblies. The test models were chosen well and demonstrate the challenge associated with chemical shift prediction.
The results show important acceleration gains achieved, and the RMSD comparison clearly indicates a successful implementation. As such this is a significant contribution to the field.
One minor suggestion is that the authors include a brief comment on how successful PPM_One is when compared to experimental NMR results (if available) for the various chosen models.

Thank you and we have added additional explanation in the paper to address this question. This addition is now available in the “Validation of Results” section (towards the end)
